# Supplementary material for: The Impact of HER2-Low Expression on Oncologic Outcomes in Hormone Receptor-Positive Breast Cancer
Source: Cancers (Basel). 2023 Nov 10;15(22):5361. doi: 10.3390/cancers15225361 (PMC10670388; doi:10.3390/cancers15225361)
Supplement: Supplementary file 1 [file cancers-15-05361-s001.zip › cancers-2699995-supplementary.pdf]

Supplementary Table S1. Clinicopathologic characteristics of postmenopausal patients according to HER2 expression

	HER2 status		
	HER2-0	HER2-low	*p* value
**Clinical variables**			
Total no.	1415	1923	
Age			
Mean±SD	59.45±8.28	59.26±7.76	0.510
Median(range)			
BRCA 1/2 mutation, no. (%)			0.009
Not detected	1409(99.6%)	1898(98.7%)	
Detected	6(0.4%)	25(1.3%)	
Breast surgery, no. (%)			0.004
BCS	1113(78.7%)	1431(74.4%)	
Mastectomy	302(21.3%)	492(25.6%)	
Axillary surgery, no. (%)			0.003
No axillary surgery	3(0.2%)	10(0.5%)	
SLNB	957(67.6%)	1391(72.3%)	
ALND	455(32.2%)	522(27.1%)	
Adjuvant chemotherapy, no. (%)			0.013
Not done	901(63.9%)	1305(68.1%)	
Yes	508(36.1%)	612(31.9%)	
Neoadjuvant chemotherapy, no. (%)			0.661
No	1367(96.6%)	1863(96.9%)	
Yes	48(3.4%)	60(3.1%)	
Radiation therapy, no. (%)			0.060
No	232(16.5%)	365(19.1%)	
Yes	1172(83.5%)	1550(80.9%)	
Anti-hormonal therapy, no. (%)			0.077
Tamoxifen	333(24.0%)	395(21.4%)	
Aromatase inhibitor	1054(76.0%)	1452(78.6%)	
**Pathologic variables**			
*T stage			0.853
1	946 (69.2%)	1267(68.0%)	
2	382 (27.9%)	543(29.2%)	

3	35(2.6%)	49(2.6%)	
4	4(0.3%)	4(0.2%)	
*N stage			0.703
0	908(66.7%)	1241(66.9%)	
1	348(25.6%)	486(26.2%)	
2	69(5.1%)	78(4.2%)	
3	36(2.6%)	49(2.6%)	
Histologic grade			<0.001
1	542	565	
2	725	1154	
3	136	194	
Nuclear grade			<0.001
1	373	273	
2	873	1433	
3	163	215	
Ki-67, %			0.482
<20%	910	1203	
≥20%	290	408	
Lymphovascular invasion			0.458
No	1051	1450	
Yes	364	473	

* 108 patients who received neoadjuvant chemotherapy were not included.
